# Acute nitric oxide synthase inhibition and endothelin-1-dependent arterial pressure elevation

**DOI:** 10.3389/fphar.2014.00057

**Published:** 2014-04-01

**Authors:** Robert M. Rapoport

**Affiliations:** Research Service, Department of Pharmacology and Cell Biophysics, Veterans Affairs Medical Center, University of Cincinnati College of MedicineCincinnati, OH, USA

**Keywords:** nitric oxide synthase inhibitor, endothelin-1, arterial blood pressure, endothelin receptor antagonist, endothelin converting enzyme inhibitor, acute

## Abstract

Key evidence that endogenous nitric oxide (NO) inhibits the continuous, endothelin (ET)-1-mediated drive to elevate arterial pressure includes demonstrations that ET-1 mediates a significant component of the pressure elevated by acute exposure to NO synthase (NOS) inhibitors. This review examines the characteristics of this pressure elevation in order to elucidate potential mechanisms associated with the negative regulation of ET-1 by NO and, thereby, provide potential insight into the vascular pathophysiology underlying NO dysregulation. We surmise that the magnitude of the ET-1-dependent component of the NOS inhibitor-elevated pressure is (1) independent of underlying arterial pressure and other pressor pathways activated by the NOS inhibitors and (2) dependent on relatively higher NOS inhibitor dose, release of stored and *de novo* synthesized ET-1, and ET_A_ receptor-mediated increased vascular resistance. Major implications of these conclusions include: (1) the marked variation of the ET-1-dependent component, i.e., from 0 to 100% of the pressure elevation, reflects the NO-ET-1 regulatory pathway. Thus, NOS inhibitor-mediated, ET-1-dependent pressure elevation in vascular pathophysiologies is an indicator of the level of compromised/enhanced function of this pathway; (2) NO is a more potent inhibitor of ET-1-mediated elevated arterial pressure than other pressor pathways, due in part to inhibition of intravascular pressure-independent release of ET-1. Thus, the ET-1-dependent component of pressure elevation in vascular pathophysiologies associated with NO dysregulation is of greater magnitude at higher levels of compromised NO.

## INTRODUCTION

Amongst the major factors which regulate arterial pressure are the vasodilator, nitric oxide (NO), and the vasoconstrictor, endothelin (ET)-1 (Lavallée et al., 2001; Bourque et al., 2011). Pressure regulation by NO and ET-1 is complex and extends beyond their individual depressor and pressor actions, respectively, due to the numerous interactions between NO and ET-1. These interactions include (1) ET-1 release of NO from the vascular endothelium, mediated by endothelial ET_B_ receptors; (2) NO inhibition of contraction to ET-1, the contraction mediated by ET_A_ and/or ET_B_ receptors (Rapoport and Zuccarello, 2012), and (3) NO inhibition of ET-1 formation/release (Lavallée et al., 2001; Bourque et al., 2011).

Not unexpectedly, the relative roles of these different ET-1-NO interactive mechanisms in the regulation of arterial pressure are not entirely clear due to the difficulty in the differentiation of the ET-1-NO interactions *in vivo*. In fact, these mechanisms are largely delineated *ex vivo* and, moreover, through the use of NO donors and exogenous ET-1 both *ex* *vivo* and *in vivo* (Lavallée et al., 2001; Bourque et al., 2011).

Although not directly addressing the differential involvement of these mechanisms in the elevation of arterial pressure, acute challenge with NO synthase (NOS) inhibitors present a unique opportunity for the assessment of the overall importance of endogenous NO in the modulation of the ET-1-mediated drive to elevate arterial pressure. That is, a component of the NOS inhibitor-elevation of arterial pressure is ET-1-mediated, as determined with ET receptor antagonists and an ET converting enzyme inhibitor (for reviews which incorporated this subject see Lavallée et al., 2001; Bourque et al., 2011).

Thus, we presently consider that (1) a detailed examination of the characteristics of the ET-1-dependent, elevated pressure due to acute challenge with NOS inhibitor may provide an *in vivo* context for mechanistic studies directed toward uncovering the intertwined NO and ET-1 pathways in the regulation of arterial pressure and (2) these characteristics would likely provide insight into the vascular pathophysiology resulting from NO dysregulation.

## ET-1 AND PRESSURE ELEVATED BY ACUTE NOS INHIBITOR

### ET CONVERTING ENZYME INHIBITION

**FIGURE 1:**
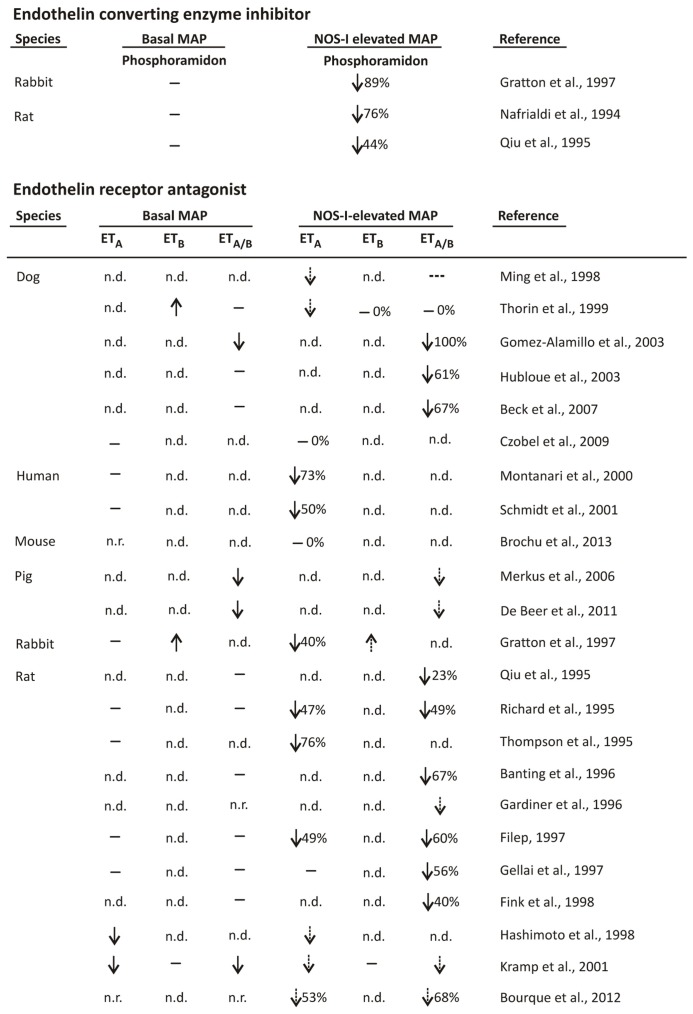
**Effects of ET converting enzyme inhibitor and ET receptor antagonist on basal and NOS inhibitor-elevated arterial pressure.** MAP = mean arterial pressure and ET_A_, ET_B_, and ET_A/B_ = ET type A, type B, and A plus B receptor antagonists, respectively. ↑, ↓, -, n.d., and n.r. signify increased, decreased, no change, not determined, and not reported, respectively. Dashed arrow and broken dash represent the directional change and lack of change, respectively, in NOS inhibitor-elevated pressure by ET receptor antagonist as compared to NOS inhibitor-elevated basal pressure in the absence of ET receptor antagonist. Percent inhibitions shown represent reported values or, if not reported, were estimates. Fink et al. (1998) utilized stroke-prone spontaneously hypertensive rat.

Phosphoramidon, an ET converting enzyme inhibitor, variably lowered the NOS inhibitor-elevated pressure (Nafrialdi et al., 1994; Qiu et al., 1995; Gratton et al., 1997; **Figure 1**). The relative magnitude of the phosphoramidon-sensitive to -insensitive component ranged from approximately half to nearly the total pressure elevated by NOS inhibitor, as determined in rabbit and rat (Nafrialdi et al., 1994; Qiu et al., 1995; Gratton et al., 1997; **Figure 1**). This variability was not due to different efficacies of phosphoramidon inhibition of ET converting enzyme in these studies since (a) in rabbit, intraventricular 10 mg/kg phosphoramidon reduced by 88% big ET-1-elevated arterial pressure (Gratton et al., 1997). Moreover, the considerable phosphoramidon inhibition of the elevated pressure due to big ET-1 occurred even though big ET-1 increased pressure by 57 mmHg in comparison to the NOS inhibitor-elevated pressure of only 17 mmHg (Gratton et al., 1997); (b) in rat, the phosphoramidon doses (intravenous 10 and 15 mg/kg/h; Nafrialdi et al., 1994; Qiu et al., 1995, respectively) were similar to those used in another rat study in which the big ET-1-elevated arterial pressure was abolished (Pollock and Opgenorth, 1991). Basal arterial pressure was also not a factor in the phosphoramidon reduction of the NOS inhibitor-elevated elevated pressure since basal pressure was not lowered by phosphoramidon (Nafrialdi et al., 1994; Qiu et al., 1995; Gratton et al., 1997; **Figure 1**).

### ET RECEPTOR ANTAGONISM

#### ET_**A**_ and ET_**A/B**_ receptor antagonist

ET_A_ and ET_A/B_ receptor antagonist also reduced NOS inhibitor-elevated pressure (Qiu et al., 1995; Richard et al., 1995; Thompson et al., 1995; Banting et al., 1996; Gardiner et al., 1996; Filep, 1997; Gellai et al., 1997; Gratton et al., 1997; Fink et al., 1998; Hashimoto et al., 1998; Ming et al., 1998; Thorin et al., 1999; Montanari et al., 2000; Kramp et al., 2001; Schmidt et al., 2001; Gomez-Alamillo et al., 2003; Hubloue et al., 2003; Merkus et al., 2006; Beck et al., 2007; Czóbel et al., 2009; de Beer et al., 2011; Bourque et al., 2012; Brochu et al., 2013; **Figure 1**). In a large majority of these studies the reduced NOS inhibitor-elevated pressure occurred in the absence of decreased basal pressure due to ET_A_/ET_A/B_ receptor antagonist (Qiu et al., 1995; Richard et al., 1995; Thompson et al., 1995; Banting et al., 1996; Filep, 1997; Gellai et al., 1997; Gratton et al., 1997; Fink et al., 1998; Thorin et al., 1999; Montanari et al., 2000; Schmidt et al., 2001; Hubloue et al., 2003; Beck et al., 2007; **Figure 1**), while in several studies ET_A_/ET_A/B_ receptor antagonist decreased basal pressure (Hashimoto et al., 1998; Kramp et al., 2001; Gomez-Alamillo et al., 2003; Merkus et al., 2006; de Beer et al., 2011; **Figure 1**). The relative magnitudes of the ET_A_/ET_A/B_ receptor antagonist-sensitive component of the NOS inhibitor-elevated pressure also varied greatly, ranging from 0 to 100% (Qiu et al., 1995; Richard et al., 1995; Thompson et al., 1995; Banting et al., 1996; Filep, 1997; Gellai et al., 1997; Gratton et al., 1997; Fink et al., 1998; Thorin et al., 1999; Montanari et al., 2000; Schmidt et al., 2001; Gomez-Alamillo et al., 2003; Hubloue et al., 2003; Beck et al., 2007; Czóbel et al., 2009; Brochu et al., 2013; **Figure 1**). The variability was not due to the use of:

(a) ET_A_ versus ET_A/B_ receptor antagonist, since both ET_A_ and ET_A/B_ receptor antagonist reduced the NOS inhibitor-elevated pressure to similar magnitudes (Richard et al., 1995; Filep, 1997; Bourque et al., 2012). Furthermore, ET_A/B_ receptor antagonist was less efficacious than ET_A_ receptor antagonist in dog (Ming et al., 1998; Thorin et al., 1999). While the greater inhibitory efficacy of ET_A_ as compared to ET_A/B_ receptor antagonist (Ming et al., 1998; Thorin et al., 1999) is not entirely clear, one possible explanation is that ET_B_ receptor antagonism may cause additional effects that limit the overall ET_A_ receptor reduction of the NOS inhibitor-elevated pressure.

It should be also be noted, however, that there is some limited evidence that non-selective ET receptor antagonism is required to fully expose the ET-1-dependent component of the NOS-I-elevated pressure. In rat, 10 mg/kg BQ123 (ET_A_ receptor antagonist) did not decrease the NOS inhibitor-elevated pressure while 10 mg/kg SB209670 (ET_A/B_ receptor antagonist) inhibited the elevation by 56% (Gellai et al., 1997). These findings raise the possibility that smooth muscle ET_B_ receptors also mediate the elevated arterial pressure, presumably through ET-1-induced vasoconstriction. It may also be considered that cross-talk between the ET_A_ and ET_B_ receptors (Rapoport and Zuccarello, 2012) is responsible for the greater inhibitory effect of ET_A/B_ versus ET_A_ receptor antagonist (Gellai et al., 1997).

(b) Different species, since amongst dog and rat studies the reductions of the NOS inhibitor-elevated pressure by ET_A/B_ receptor antagonists both ranged considerably, i.e., from 0 to 100% (Thorin et al., 1999; Gomez-Alamillo et al., 2003; Hubloue et al., 2003; Beck et al., 2007) and 23–67% (Qiu et al., 1995; Richard et al., 1995; Banting et al., 1996; Filep, 1997; Gellai et al., 1997; Fink et al., 1998), respectively (**Figure 1**).

(c) Conscious versus anesthetized animals, since in conscious and anesthetized rat, ET_A/B_ receptor antagonist decreased NOS inhibitor-elevated arterial pressure by 23–67% (Qiu et al., 1995; Banting et al., 1996; Filep, 1997; Gellai et al., 1997) and by 40% and 49% (Richard et al., 1995; Fink et al., 1998), respectively (**Figure 1**).

(d) Different doses of ET receptor antagonist, since ET receptor antagonist doses were generally highly efficacious. Specifically, in several studies in which the effects of ET receptor antagonist on both NOS inhibitor and exogenous ET-1- and big ET-1-elevated pressure were examined, the ET receptor antagonist greatly inhibited the elevated pressure due to ET-1/big ET-1 even though the ET-1/big ET-1 dose generally induced a greater increase in pressure than the NOS inhibitor (Thompson et al., 1995; Filep, 1997; Gratton et al., 1997; Fink et al., 1998; Hubloue et al., 2003; Beck et al., 2007). However, an untested (to our knowledge) assumption underlying this comparative analysis of the inhibitory efficacy of ET receptor antagonist toward the NOS inhibitor- and ET-1-/big ET-1-elevated pressure is whether ET receptor antagonist efficacy is reduced by cellular events associated with NOS inhibition.

The possibility that in some studies the dose of ET receptor antagonist lacked sufficient efficacy should also be considered. For example, 2 mg/kg BQ123 failed to reduce the NOS inhibitor-elevated pressure in mouse (Brochu et al., 2013). Whether a greater BQ123 dose would have reduced the NOS inhibitor-elevated pressure (Brochu et al., 2013) remains a possibility since in rabbit, in which the NOS inhibitor-elevated pressure peaked and then decreased somewhat to a maintained level, 10 mg/kg but not 1 mg/kg BQ123 reduced the peak pressure elevation (Gratton et al., 1997). Also, in rat 10 mg/kg BQ123 failed to reduce the NOS inhibitor-elevated pressure (Gellai et al., 1997), although in another study 3 mg/kg BQ123 decreased the elevated pressure by 49% (Richard et al., 1995).

(e) Different doses of NOS inhibitor. Indeed, at successively greater NOS inhibitor doses, which induced greater magnitudes of pressure elevation, ET receptor antagonist caused increasingly larger percent inhibitions of NOS inhibitor-elevated pressure (Richard et al., 1995; Filep, 1997; Beck et al., 2007). That is, the relative ratio of the ET-1-dependent to -independent components of the NOS inhibitor-elevated pressure increased with NOS inhibitor dose and also, therefore, with greater amounts of NOS inhibitor-induced pressure elevation (Richard et al., 1995; Filep, 1997; Beck et al., 2007). The dose range of the NOS inhibitor, *N*^ω^-nitro-L-arginine methyl ester (L-NAME, intravenous bolus), in rat was 0.1–3 mg/kg (Richard et al., 1995) and 0.125–2 mg/kg (Filep, 1997), with maximal increased mean arterial pressure achieved at 1 mg/kg. In dog, the dose range of L-NAME (intravenous bolus) was 0.3–10 mg/kg, with the increase in mean arterial pressure achieved at 10 mg/kg only slightly greater than that at 3 mg/kg (Beck et al., 2007).

However, similar maximally effective (pressure elevation) doses of L-NAME established in these studies (Richard et al., 1995; Filep, 1997; Beck et al., 2007) were also used in a number of studies, i.e., 2 mg/kg (Brochu et al., 2013) and 10 mg/kg (Qiu et al., 1995; Gratton et al., 1997; Fink et al., 1998; Kramp et al., 2001). Furthermore, while other studies used NOS inhibitors other than L-NAME, i.e., *N*^ω^-monomethyl-L-arginine (L-NMMA) and *N*^ω^-nitro-L-arginine (L-NNA), these inhibitors were used at similar or even greater doses than in the studies with L-NAME. Specifically, L-NMMA was administered at 30 mg/kg (Gardiner et al., 1996) and 6 mg/kg followed by 3.6 mg/kg/h infusion (in human; Schmidt et al., 2001), and L-NNA was administered at 4 mg/kg (Czóbel et al., 2009), 5 mg/kg (Hashimoto et al., 1998), 10 mg/kg (Gellai et al., 1997), 20 mg/kg (Merkus et al., 2006; de Beer et al., 2011), and 5 mg/kg followed by 5 mg/kg/h infusion (Hubloue et al., 2003).

It should be noted that NOS inhibitor was also infused in the absence of prior intravenous bolus. In animals, L-NAME was infused over the dose range of 3–12 mg/kg/h (Nafrialdi et al., 1994; Thompson et al., 1995; Ming et al., 1998; Thorin et al., 1999; Gomez-Alamillo et al., 2003). In contrast to another human study (Schmidt et al., 2001, see above), only a relatively low dose of L-NAME was infused, i.e., 0.18 mg/kg/h (Montanari et al., 2000). Finally, in two rat studies, L-NAME was injected i.p. at 100 mg/kg (Banting et al., 1996; Bourque et al., 2012).

#### ET_**B**_ receptor antagonist

The effect of ET_B_ receptor antagonist on the NOS inhibitor-elevated pressure was difficult to evaluate in some studies due to the increased basal pressure (Gratton et al., 1997; Thorin et al., 1999; **Figure 1**). In these studies, ET_B_ receptor antagonist failed to alter the NOS inhibitor-elevated pressure (Thorin et al., 1999) or the pressure was possibly increased (Gratton et al., 1997). In the one study in which ET_B_ receptor antagonist did not alter basal arterial pressure, NOS inhibitor-elevated pressure remained unaltered by the ET_B_ receptor antagonist (Kramp et al., 2001).

### ET-1 AND BIG ET-1 PLASMA LEVELS

ET-1 plasma levels were inconsistently elevated by NOS inhibitors. Although elevated ET-1 plasma levels were detected following acute challenge with NOS inhibitor in the dog, human, and rat, the increases were of relatively small magnitude (Richard et al., 1995; Ahlborg and Lundberg, 1997; Filep, 1997; Czóbel et al., 2009). ET-1 plasma levels were not elevated following NOS inhibitor in stroke-prone spontaneously hypertensive rat, rabbit, and conscious sheep (Tresham et al., 1994; Gratton et al., 1997; Fink et al., 1998). Also, in the human, while maximal ET-1 plasma levels and arterial pressure were observed 20 min and 10 min (initial recording) post NOS inhibitor, respectively, ET-1 plasma levels were not elevated at 30 min post NOS inhibitor even though the pressure remained elevated (Ahlborg and Lundberg, 1997).

A limited temporal association between elevated ET-1 plasma levels and NOS inhibitor-elevated arterial pressure was observed in the dog since both elevated ET-1 plasma levels and elevated pressure were observed after 15 min infusion with NOS inhibitor (Czóbel et al., 2009). On the other hand, ETR-P1/fl peptide, an ET_A_ receptor antagonist which purportedly also binds ET-1 (Baranyi et al., 1995, 1998), completely prevented the increased ET-1 plasma levels but did not significantly reduce and only partially reduced NOS inhibitor-elevated pressure and -elevated peripheral vascular resistance, respectively (Czóbel et al., 2009).

The inability to detect NOS inhibitor-elevated ET-1 plasma levels may be due to clearance of plasma ET-1 by ET_B_ receptors located in the lung and other tissues (de Nucci et al., 1988). However, elevated ET-1 plasma levels were still not detected in the rabbit when ET_B_ receptor antagonist was added 10 min after bolus injection of NOS inhibitor (Gratton et al., 1997). Although, the detection of elevated ET-1 plasma levels in response to NOS inhibitor in stroke-prone spontaneously hypertensive rat was dependent on the presence of an ET_A/B_ receptor antagonist (Fink et al., 1998). An additional factor that undoubtedly complicates the detection of elevated plasma ET-1 levels is plasma dilution.

Big ET-1 plasma levels were elevated by intravenous bolus NOS inhibitor in the rabbit (Gratton et al., 1997). The increase was transient, with elevated levels at 1 and 2 min which returned to basal by 10 min (Gratton et al., 1997). Interestingly, ET_B_ receptor antagonist also elevated big ET-1 levels and, furthermore, NOS inhibitor prevented this elevation (Gratton et al., 1997).

## TIME COURSE OF ELEVATED PRESSURE BY ACUTE NOS INHIBITOR

The differential effects of ET receptor antagonist and phosphoramidon on the phases of NOS inhibitor-elevated pressure suggest a dependency on different pools of ET-1 (see Conclusion/speculations). That is, (a) NOS inhibitor elicited an initial rapid (minutes) pressure elevation followed by a plateau phase, as demonstrated with intravenous bolus NOS inhibitor in mouse (Brochu et al., 2013), rat (Gardiner et al., 1996; Fink et al., 1998), and rabbit (Gratton et al., 1997), and intraperitoneal NOS inhibitor in rat (Banting et al., 1996) and (b) ET receptor antagonist reduced both the initial rapid phase and plateau phase of NOS inhibitor-elevated pressure in rat and rabbit (Banting et al., 1996; Gardiner et al., 1996; Gratton et al., 1997). It should be noted, however, that ET_A/B_ receptor antagonist did not reduce the initial rapid phase in the stroke-prone spontaneously hypertensive rat (Fink et al., 1998). Whether the underlying effects of stroke/hypertension resulted in the apparent ET-1 independence of the initial phase (Fink et al., 1998) should be considered.

Phosphoramidon (10 mg/kg), in contrast to ET receptor antagonist, did not reduce the rapid phase of NOS-elevated pressure in rabbit (Gratton et al., 1997). However, the plateau phase of the pressure elevation was inhibited by phosphoramidon (Gratton et al., 1997).

## INFLUENCE OF PRESSURE/OTHER PRESSOR PATHWAYS

NOS inhibitor-elevated pressure in rat was decreased by combined vasopressin_1/2_, angiotensin_1_, and alpha adrenergic receptor antagonists (Banting et al., 1996), ganglionic blockade and pithing (Richard et al., 1995), and epidural lidocaine anesthesia (Beck et al., 2007), while the magnitude of the ET-1-dependent component of the NOS inhibitor-elevated pressure remained unaltered. Indeed, the absolute mmHg reduced by ET receptor antagonist following ganglionic blockade and pithing was greater than in untreated rats (Richard et al., 1995). Also of possible relevance is that ET receptor antagonist did not decrease the elevated arterial pressure due to angiotensin in rabbit (Gratton et al., 1997) and phenylephrine in rat (Richard et al., 1995).

The effect of ET receptor antagonist challenge prior versus during NOS inhibitor-elevated pressure also reflects differences in pressure elevation. Although numerous studies investigated the effects of ET receptor antagonist on the NOS inhibitor-elevated pressure by antagonist addition prior to NOS inhibitor (Richard et al., 1995; Banting et al., 1996; Filep, 1997; Gellai et al., 1997; Fink et al., 1998; Hashimoto et al., 1998; Montanari et al., 2000; Kramp et al., 2001; Gomez-Alamillo et al., 2003; Hubloue et al., 2003; Beck et al., 2007; Czóbel et al., 2009; Brochu et al., 2013; **Figure 1**), or during the NOS inhibitor plateau pressure elevation (Thompson et al., 1995; Banting et al., 1996; Gardiner et al., 1996; Ming et al., 1998; Thorin et al., 1999; Kramp et al., 2001; Schmidt et al., 2001; Merkus et al., 2006; de Beer et al., 2011; **Figure 1**), only two studies performed both protocols (Banting et al., 1996; Kramp et al., 2001). Similar magnitudes of reduction of the NOS inhibitor-elevated pressure were observed in rat when ET receptor antagonist was added prior to NOS inhibitor as compared to during the NOS inhibitor plateau pressure elevation (Banting et al., 1996). Furthermore, similar time periods (~5 min) were required to achieve plateau pressure elevation following ET receptor antagonist addition prior to NOS inhibitor and for ET receptor antagonist to reverse the NOS inhibitor plateau elevation and elicit a lower level of plateau pressure elevation (Banting et al., 1996). While another rat study also determined the effects of ET receptor antagonist challenge prior to NOS inhibitor and during the elevated pressure due to NOS inhibitor, quantification of the reductions of the NOS inhibitor-elevated pressure was difficult due to the lowered basal arterial pressure by the ET receptor antagonist (Kramp et al., 2001).

## INCREASED VASCULAR RESISTANCE AND ET RECEPTOR ANTAGONIST

While there is considerable evidence that ET receptor antagonist inhibits the increased peripheral vascular resistance due to NOS inhibitor, studies in which the effects of ET receptor antagonist on both the increased peripheral vascular resistance and on blood pressure have not been surmized. Indeed, ET receptor antagonist caused similar percent reductions of NOS inhibitor-elevated pressure and -elevated peripheral vascular resistance in anesthetized rat (Thompson et al., 1995) and dog (Gomez-Alamillo et al., 2003). Also in anesthetized dog, ET receptor antagonist reduced NOS inhibitor-elevated peripheral vascular resistance by as much as 60% (Czóbel et al., 2009). Although in this study (Czóbel et al., 2009) the ET receptor antagonist reduction of the NOS inhibitor-elevated pressure was not statistically significant, this lack of significance may be attributed to the relatively large variability in the pressure measurements.

In possible contrast, in conscious dog, ET receptor antagonist decreased NOS inhibitor-elevated pressure by 67% while, unexpectedly, peripheral vascular resistance was not decreased (Beck et al., 2007). While the lack of ET receptor antagonist reduction of the NOS inhibitor-elevated peripheral vascular resistance might suggest the involvement of other hemodynamic factors, an effect on NOS inhibitor-induced decreased cardiac output was apparently not observed (Beck et al., 2007).

Interestingly, in this same study (Beck et al., 2007) but with dogs subjected to epidural anesthesia, ET receptor antagonist decreased the NOS inhibitor-elevated peripheral vascular resistance and the elevated arterial pressure by 50 and 67%, respectively.

## CONCLUSION/SPECULATIONS

**FIGURE 2:**
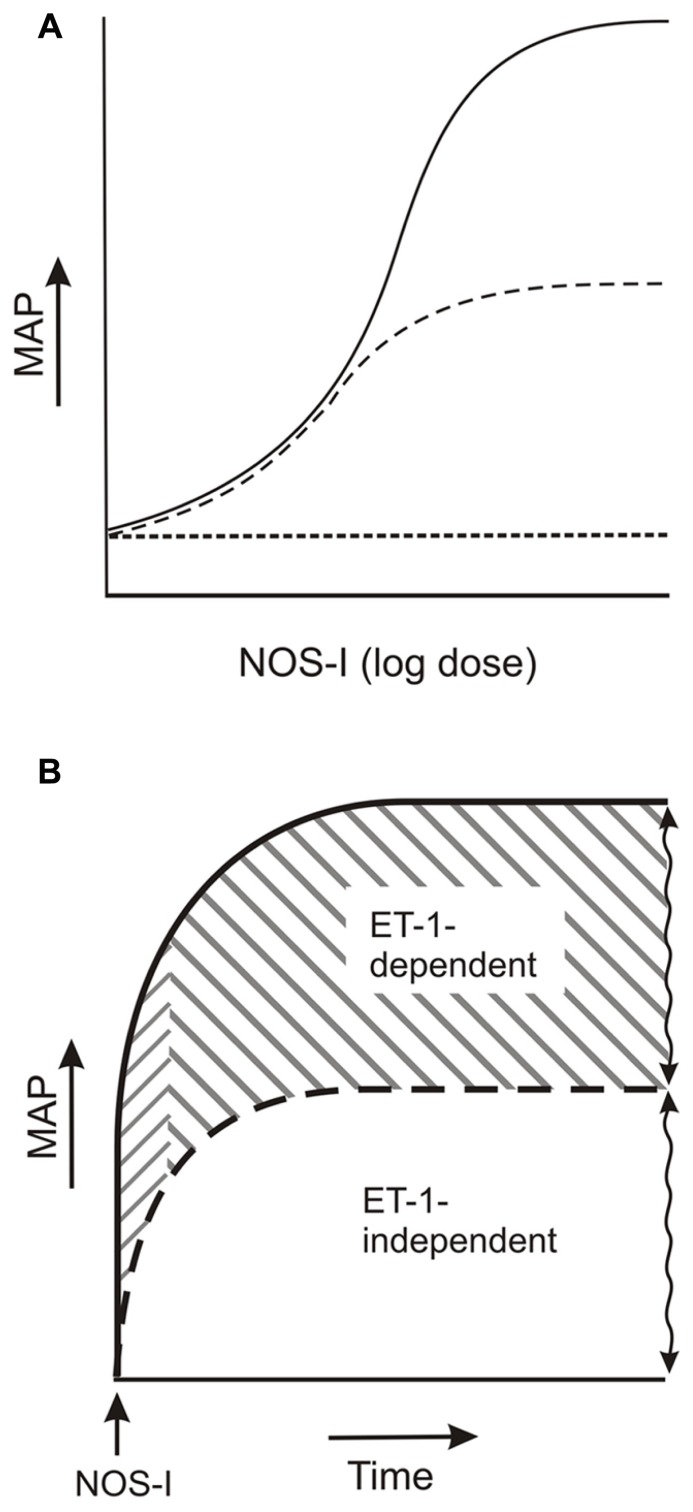
**ET-1-dependent and -independent components of the elevated arterial pressure due to acute NOS inhibitor.** **(A)** Dose-response: NOS inhibitor (NOS-I) dose and ET-1-independent (dashed line) and -dependent pressure elevation (solid line). **(B)** ET-1 pools: ET-1-dependent component (solid line) consists of an initial rapid and a subsequent plateau increase in pressure (rightward upward and rightward downward slanted lines, respectively), which reflect dependencies on stored ET-1 and *de novo* synthesized ET-1 pools, respectively. ET-1-independent pressure elevation is indicated by the dashed line. Squiggled vertical lines indicate varied magnitudes of ET-1-independent and -dependent components of pressure elevation. See text for further details.

As illustrated in the model of **Figure 2**, considerable evidence from studies on the effects of phosphoramidon and ET_A_ and ET_A_/ET_A/B_ receptor antagonists on the elevated pressure due to acute challenge with NOS inhibitor demonstrates that ET-1 mediates a component of the pressure elevation. This component demonstrates marked variability which cannot be accounted for by numerous experimental parameters. Also, the component is independent of other NOS inhibitor-elevated pressure pathways (see also Lavallée et al., 2001; Bourque et al., 2011). Thus, the variation reflects the overall capacity of the NO-regulated, ET-1-mediated pathways to elevate pressure.

Another characteristic of the ET-1-dependent component of the NOS inhibitor-elevated pressure is the apparent dependency of the pressure elevation on different ET-1 pools, i.e., the initial rapid phase and plateau phase of pressure elevation may reflect ET-1 release from stored ET-1 and from *de novo* synthesized ET-1, respectively. It is important to note that these observations provide at least indirect *in vivo* evidence for the involvement of increased ET-1 release in the NOS inhibitor-elevation of arterial pressure. Indeed, direct support for increased ET-1 release by NOS inhibitor as evidenced by elevated plasma ET-1 levels is inconsistent, presumably due to ET-1 clearance and plasma dilution. Thus, while enhanced ET-1 contraction due to removal of NO-mediated vasodilatation undoubtedly contributes to the NOS inhibitor-elevated arterial pressure (Lerman et al., 1992; Xu et al., 2001; Hocher et al., 2004), ET-1 release represents a contributing factor.

With respect to the mechanism underlying the increased ET-1 release, several lines of evidence suggest that the release is not the result of elevated pressure. Indeed, this conclusion also infers that the component of the NOS inhibitor-elevated pressure attributed to reversal of NO relaxation of smooth muscle (Banting et al., 1996) also does not trigger ET-1 release. First, lowered basal pressure due to inhibition of a number of pressor pathways did not decrease the magnitude of the ET-1-dependent component of NOS inhibitor-elevated pressure. Second, ET receptor antagonist caused a similar magnitude of inhibition of the NOS inhibitor-elevated pressure when added prior to NOS inhibitor addition and during the plateau pressure elevation. Third, ET receptor antagonist did not reduce the pressure elevated by angiotensin II and phenylephrine (Richard et al., 1995; Gratton et al., 1997). It should also be considered; however, that the lack of effect of ET receptor antagonist on pressure due to these agents (Richard et al., 1995; Gratton et al., 1997) resulted from increased NO formation, which prevented ET-1 release. It would be of interest to investigate, therefore, whether ET receptor antagonist reduction of NOS inhibitor-elevated pressure is increased in the presence of a pressor agent other than ET-1.

In any case, the mechanism whereby *de novo* synthesized ET-1 is released may be due to reversal of NO inhibition of the conversion of big ET-1 to ET-1, since NOS inhibitor elevated big ET-1 plasma levels (Gratton et al., 1997). Furthermore, this regulatory pathway appears to be shared by ET_B_ receptor-mediated formation of NO, since NOS inhibitor prevented the ET_B_ receptor-mediated increase in big ET-1 plasma levels (Gratton et al., 1997).

## THERAPEUTIC IMPLICATIONS

Based on the independence of the ET-1-dependent component of the elevated pressure due to acute challenge with NOS inhibitor from other pressor systems, it is reasonable to conclude that the ET-1-dependent effects of NOS inhibition specifically reflect NO-ET-1 regulatory pathways. Thus, for example, ET receptor antagonist partial prevention of the acute NOS inhibitor-elevated pressure in high-salt diet induced hypertension in bradykinin_2_ receptor knockout but not wild type mouse suggests an enhancement of the NO-ET-1 regulatory pathway (Brochu et al., 2013; **Figure 1**). Also, acute hypoxia may depress the NO-ET-1 regulatory pathway since hypoxia lowered the magnitude of the NOS inhibitor-elevated, ET-1-dependent pressure from 61 to 40% (Hubloue et al., 2003; **Figure 1**).

Additionally, at least based on a limited number of studies in which ET_A_ and ET_A_/ET_A/B_ receptor antagonists were compared head-to-head, the elevated pressure due to NOS inhibitor appears to result from ET-1 activation of smooth muscle ET_A_ receptors. Thus, ET_A_ receptor antagonist should effectively reduce the increased blood pressure that may involve, e.g., endothelial ET_B_ receptor dysfunction. On the other hand, an ET_A/B_ receptor antagonist may possess greater therapeutic efficacy in pathologies in which ET_B_ receptor activation contributes significantly to the pressure elevation.

It should also be noted that the endogenous NOS inhibitor, asymmetric dimethylarginine, has been implicated in numerous vascular pathologies (Arrigoni et al., 2010; Tamás et al., 2013; Worthmann et al., 2013). Moreover, these pathologies include conditions in which plasma asymmetric dimethylarginine levels rise relatively rapidly, such as pre-eclampsia and acute stroke (Arrigoni et al., 2010; Tamás et al., 2013; Worthmann et al., 2013). Thus, the *in vivo* effects of acute NOS inhibitor are highly relevant with respect to investigations into the altered NO-ET-1 regulatory pathways in these pathologies.

Finally, based on the observations that the ET-1-dependent component of the NOS inhibitor-elevated pressure increase with NOS inhibitor dose (Richard et al., 1995; Filep, 1997; Beck et al., 2007), it is of interest to speculate that ET receptor antagonist treatment of pathologies associated with NO dysregulation is more effective under conditions of greater dysregulation. Whether this greater efficacy reflects increased relative involvement of ET-1 release or enhanced ET-1-induced vasoconstriction requires additional understanding of the mechanism whereby NO regulates these pathways.

## Conflict of Interest Statement

The authors declare that the research was conducted in the absence of any commercial or financial relationships that could be construed as a potential conflict of interest.
